# A Case-Control Study of TP53 R72P Polymorphism in the Breast Cancer Patients of Ethnic Kashmiri Population

**DOI:** 10.4021/wjon261w

**Published:** 2011-01-01

**Authors:** Nidda Syeed, A. Syed Sameer, Safiya Abdullah, Syed Akhtar Husain, Mushtaq A Siddiqi

**Affiliations:** a Department of Immunology & Molecular Medicine, SKIMS, Srinagar, Kashmir, India; b Department of Biosciences, Jamia Millia Islamia, New Delhi, India; d These authors contributed equally

**Keywords:** Breast cancer, Tp53 polymorphism, Kashmir, R72P

## Abstract

**Background:**

TP53 R72P polymorphism has been proposed as a risk factor for breast cancer and is more likely to differ among different ethnic populations. We carried out the study to determine the role of R72P polymorphism in breast cancer patients of Kashmir, an ethnic population by PCR-RFLP.

**Methods:**

To evaluate the role of this polymorphism in our ethnic Kashmiri population, we devised our study to study its role in breast cancer patients and healthy controls. The contribution of TP53 R72P polymorphism in 130 breast cancer patients and 220 female healthy controls was assessed using a PCR-RFLP (polymerase chain reaction-restriction fragment length polymorphism).

**Results:**

We observed that women with PP genotype have increased risk for developing breast cancer. We found Pro/Pro genotype statistically significantly associated with dwelling, lymph node metastases, histopathological grade, and menopausal status. Pro/Pro genotype in cases and controls was observed and it was found that it is significantly associated with the breast cancer.

**Conclusions:**

Our findings suggest that TP53 R72P polymorphism is a risk factor in breast cancer. Furthermore, these results suggest Pro72 allele is associated with higher risk for breast cancer patients. Women with PP genotype have increased risk for developing breast cancer.

## Introduction

Breast cancer is one of the most common malignancies in women. It continues to be a major burden and cause of death among women worldwide. Worldwide, breast cancer is by far the most common cancer amongst women, with an incidence rate more than twice that of colorectal cancer and cervical cancer and about three times that of lung cancer. According to BIC database, majority of high risk families of different ethnicities, the germline mutations are scattered throughout both genes and are family specific.

Kashmir valley is located in the northernmost part of India. The Kashmir valley lies between Himalayas and the Pir Panjal Range, about 5000 ft from the sea level. The genetic makeup of the Kashmiri population is preserved due to consanguineous marriage that has lead to the preservation of the genetics of this population. The people of Kashmir constitutes a different set of dietary habits that have high percentage of nitroso compounds, amines and nitrates present in local food stuff [1-3] that consists of dried and smoked fish and meat, dried and pickled vegetables, red chilli, Hakh (a leafy vegetable of Brassica family), hot noon chai (salted tea) and Hukka (water pipe) smoke.

The TP53, a tumor suppressor gene plays a key role in preservation of genome integrity. The TP53 gene has been found mutated in various cancers including breast cancer [4], indicating its importance in cancer development, acting as a checkpoint control for recognizing damaged DNA and inducing repair or apoptosis [5]. Apart from the studies that have been carried out on the mutational status of TP53, there are many studies regarding the importance of the genetic polymorphisms of this gene. One of the important polymorphisms of TP53 gene occurs at codon 72 that encodes either proline or an arginine residue. TP53 protein with proline residue is structurally different from the one with arginine, and has functionally different role in inducing both apoptosis and cell cycle repair, in addition to affecting the function of somatic TP53 sequence variants [6, 7]. The arginine (arg) to proline change at codon 72 is located in the proline-rich region of the protein and affects the structure of the putative SH3-binding domain. These two variant protein forms behave differently: while the Arg/Arg genotype has been reported to induce apoptosis more effectively than the Pro/Pro genotype [7, 8], the Pro/Pro genotype appears to induce a higher level of G1 arrest than the Arg/Arg genotype [9, 10]. It has been seen that breast cancer patients with the Pro/Pro genotype of TP53 have poorer survival than those with other genotypes [11]. In addition, retention of the Arg allele of TP53 in tumor tissue samples of Arg/Pro heterozygous breast cancer patients has been associated with reduced disease-free and overall survival [12]. On the basis of this literature, we designed our study to determine the TP53 codon 72 genotype related to disease-free survival and overall survival of breast cancer affected patients.

## Materials and Methods

### Breast cancer patients

All breast cancer patients included in the study were both male and female, with the histopathological diagnosis of the breast cancer. The patient participation was obtained through informed consent and after approval from the Ethics Committee of Sher-I-Kashmir Institute of Medical Sciences.

A cohort of 130 breast cancer tissue samples were collected consisting of tumor tissues and adjacent normal tissue. Only the tissue samples confirmed by histopathological studies to be cancerous were included in the study.

### Controls

Patients attending the Department of general medicine at Sher-I-Kashmir Institute of Medical Sciences (SKIMS) for general check up were screened. A total of 260 patients visited the SKIMS; out of 260 patients, only 220 agreed to take part in the present study, henceforth written informed consent was obtained from all patients for their participation.

### R72P gene polymorphism

The exon 4 polymorphic site of TP53 codon 72 was detected by restriction fragment length polymorphism of PCR-amplified fragments. PCR reactions were carried out in a final volume of 25 µL containing 50 ng genomic DNA template, 1 x PCR buffer (Biotools) with 2 mM MgCl_2_, 0.4 µM of each primer (Genescript), 50 µM dNTPs (Biotools), and 0.5 U DNA polymerase (Biotools). For PCR amplification, the standard program was used as follows: an initial denaturation step at 94°C for 7 min was fol­lowed by 35 denaturation cycles of 30 s at 94°C, 30 s of annealing at 54°C, and 30 s of exten­sion at 72°C, and at last followed by a final elongation cycle at 72°C for 5 min. The amplified products (279bp) were detected on 2% agarose gel, along with a 100-bp DNA ladder. The 279-bp PCR products (20_l) were digested for 16 h at 37°C with 1 unit of *Bst*UI (Fermentas Inc, Vilnius, Lithuania); Arg/Arg wild produced two bands (160 and 119 bp); the Pro/Pro variant was identified by a single band (279 bp); and heterozygous Pro/Arg variant displayed three bands (279, 160, and 119 bp). The detection of the different alleles was carried out by horizontal ethidium bromide 4% agarose gel electrophoresis, along with a 100-bp DNA ladder.

### Statistical analysis

Observed frequencies of genotypes in breast cancer patients were compared to con­trols using chi-square or Fisher exact tests when expected frequencies were small. The chi-square test was used to verify whether genotype distributions were in Hardy-Weinberg equilibrium. Statistical significance was set at P value less than 0.05. Statistical analyses were performed with PASW version 18 Software.

## Results

We determined the TP53 codon 72 genotype in 130 breast cancer patients: 22.3% (29 of 130) of the patients were homozygous for R/R variant, 28.47% (37 of 130) were heterozygous for R/P and 49.2% (64 of 130) were homozygous for P/P variant. Whereas the pattern of TP53 R72P polymorphism in 220 healthy controls, 16.3% (36 of 220) of the patients were homozygous for R/R variant, 48.6% (107 of 220) were heterozygous for R/P, and 30.4% (67 of 220) were homozygous for P/P variant.

Our study consisted of 130 breast cancer patients, out of which 113 patients were below 50 years of age and 17 were of 50 years of age or more. Most of our patients (72) had grade I and II tumor, and 58 had grade III and IV tumor status. One hundred and five breast cancer patients had widely or moderately differentiated histopathological grade and 25 had poorly differentiated histopathological grade. Women aged 50 years or over formed the greater part of the breast cancer cases. Table 1 shows clinico-pathological characteristics related to the RR, RP and PP variants. A larger number of patients included in our study presented stages I and II than those who presented stages III and IV. Another clinical aspect investigated in this study was the relation between disease survival and Arg and Pro genotypes.

**Table 1:** Association Between TP53 Gene Codon 72 and Clinico-pathologic Characteristics

Variables	Cases (n = 130)				
	Total N = 130	RR 29 (22.3%)	R/P 37 (28.4%)	PP 64 (49.2%)	*P Value; χ*^2^
Dwelling					
Rural:	45 (34.6%)	08 (17.77%)	12 (26.66%)	25 (55.55%)	0.52 ; 1.27
Urban:	85 (65.4%)	21 (24.70%)	25 (29.41%)	29 (34.11%)	
Smoking Status					
Never:	93 (71.5%)	18 (19.35%)	27 (29.03%)	48 (51.61%)	0.42 ; 1.69
Ever:	37 (28.5%)	11 (29.72%)	10 (27.02%)	16 (43.24%)	
Menopausal Status					
Pre:	36 (27.7%)	12 (33.33%)	12 (33.33%)	12 (33.33%)	0.04 ; 6.16
Post:	94 (72.3%)	17 (18.08%)	25 (26.59%)	54 (57.44%)	
Nodal Status					
Involved	34 (26.2%)	12 (35.29%)	09 (26.47%)	13 (38.23%)	0.09 ; 4.68
Not Involved	96 (73.8%)	17 (17.70%)	28 (29.16%)	51 (53.12%)	
Tumor Stage					
II(a+b)	72 (55.4%)	19 (26.38%)	25 (34.72%)	28 (38.88%)	0.03 ; 6.93
III(a+b)+ IV	58 (44.6%)	10 (17.24%)	12 (20.68%)	36 (62.66%)	
Histopathological Tumor					
Grade					
PD	25 (19.23%)	08 (32.0%)	08 (32.0%)	09 (36.0%)	0.04 ; 6.21
MD + WD	58 + 47 (80.76%)	21 (20.0%)	29 (27.61%)	55 (52.38%)	

The codon 72 PP genotype was statistically significantly associated with the menopausal status, tumor stage, lymph node involvement and histopathological grade (Table 1). The genotype was not statistically significantly associated with the mean age at the time of diagnosis, dwelling or smoking status.

In this study we observed genotype frequencies in cases and controls were in Hardy-Weinberg equilibrium. The genotype frequencies of TP53 R72P in cases and controls were observed and it was found that PP genotype is significantly associated with the breast cancer cases (P value = 0.001) Table 2.

**Table 2:** Genotype Frequencies of TP53 Gene Codon 72 Polymorphism in Cases and Controls and Their Associations With the Risk of Breast Cancer

TP53 R72P Genotype	Cases (n = 130)	Controls (n = 220)	*OR (95% CI ); p value*
**RR**	29 (22.31%)	46 (20.91%)	1 (Ref)
**RP**	37 (28.46%)	107 (48.64%)	0.548 (0.30-0.99); 0.06
**PP**	64 (49.23%)	25 (11.36%)	4.06 (2.10-7.82); 0.00002
**RP+PP**	101 (77.69%)	132 (60.0%)	1.21 (0.71-2.06); 0.50

## Discussion

Breast cancer is the most frequent neoplasm affecting women all over the world and is considered to be the third most common malignancy in the world [13] with more than 1 million women diagnosed with breast cancer each year [14]. Breast cancer has been associated with a variety of risk factors [15] of both genetic and epigenetic changes [16].

Kashmir is a small valley present at a high altitude, with mostly consanguineous marriages resulting in preservation of the genetic pool. In addition, the unique dietary habit also results in predisposition to certain cancers including breast cancer. As has been reported earlier, breast cancer is a heterogeneous disease, and many risk factors are responsible in the development of breast cancer [17, 18]. It has been put forward that the frequency of TP53 codon-72 polymorphism varies according to ethnic group; hence we performed the study to analyze the role of TP53 R72P polymorphism in breast cancer patients in our ethnic population. The main aim of our study was to predict the predisposition towards the development of breast cancer due to single nucleotide polymorphism in TP53 codon 72.

TP53 is a classic tumor suppressor gene mutated in majority of human cancers including breast cancer. The main functions of TP53 are arresting cellular proliferation in response to various cellular stresses, including activated oncogenes, DNA damage, and hypoxia. As has been stated earlier [19], different ethnic populations express different TP53 polymorphisms and are selectively regulated; and it has also been observed that the R allele is activated during cancer development in Asians.

It has been shown by some studies [8] that TP53 Pro72 variant induces transcription activation more efficiently than TP53 Arg72 variant, and other studies have shown that TP53 Arg72 variant is more efficient in inducing apoptosis [7]. Few others showed TP53 Pro72 variant induces cell cycle arrest better than Arg72 [10]. In addition, to apoptosis and cell cycle control, p53 protein seems to be crucial in the regulation of the different DNA repair pathways [20]. A recent study revealed the ability of TP53 R72P in repairing the DNA damage, much more efficiently than the Arg72 variant expressing cells [21].

We included a total of 130 breast cancer patients; 22.3% (29 of 130) of the patients were homozygous for R/R variant, 28.47% (37 of 130) were heterozygous for R/P and 49.2% (64 of 130) were homozygous for P/P variant. Whereas the pattern of TP53 R72P polymorphism in 220 healthy controls, 16.3% (36 of 220) of the patients were homozygous for R/R variant, 48.6% (107 of 220) were heterozygous for R/P, and 30.4% (67 of 220) were homozygous for P/P variant. As has been stated earlier, [19] in Asians, R allele is more activated during cancer development, but in our study we found the P allele is much involved in the development and progression of breast cancer.

After the initial report of a statistically significantly increased risk of breast cancer in women who were homozygous for the proline allele [22], it has been found that PP variant leads to increased breast cancer risk [23], and it has also been found that R72 form of wild-type p53 is almost five times more efficient in apoptosis induction than the P72 form, which is presumed to rely on the increased localization of the R72 form of TP53 in the mitochondria when compared with the P72 form [7].

We found a significant association of PP variant with certain clinico-epidemiological, as shown in Table 1.

We found a significant association between PP variant and pre-menopausal status, suggesting that pre-menopauses of breast cancer patients are more susceptible to breast cancer development and progression.

An increased association was found between the PP variant and rural dwelling, which depicts that in our population patients from rural areas are at higher risk than the urban ones, which might be because of their diet which are rich in nitrosamines that are supposed to lead to breast cancer [24].

We also found an increased association between the PP variant and involvement of lymph nodes, which might be due to the reason that PP variant might facilitate in stimulating axillary lymph node metastasis, which is in agreement with previously functional studies in the biological consequences of these variations in P53 protein functions [8].To date axillary lymph node status is the best prognostic indicator in breast cancer [25, 26]. Therefore, present study might also act as a marker to indicate prognosis of breast cancer.

We also found a significant association between PP variant and tumor grade III and IV, which might suggest its role in harboring a higher malignant behavior.

A significant correlation was found between the PP variant and poorly differentiated histopathological grade as has been reported earlier [27], suggesting that TP53 germline and somatic mutations might be involved in different mechanisms and possibly also in different stages of tumor development.

We also found that the genotype frequencies of R72P in cases and controls, where PP genotype was significantly associated with the breast cancer cases (P value <0.05), suggested that the breast cancer patients carrying a PP allele are at much higher risk for breast cancer.

### Conclusion

We could conclude that in our population the PP variant might contribute to the development and progression of breast cancer. Two of the largest studies done of the polymorphism with breast cancer risk include more than 1000 subjects, however, no association of this polymorphism with breast cancer risk was reported [28, 29]. But in our population we suppose that it might be due to our environmental conditions and dietary habits that contribute for being at greater risk.
